# Supplementary material for: Peptidomics Unveils Distinct Acetylation Patterns of Histone and Annexin A1 in Differentiated Thyroid Cancer
Source: Int J Mol Sci. 2023 Dec 27;25(1):376. doi: 10.3390/ijms25010376 (PMC10778789; doi:10.3390/ijms25010376)
Supplement: Supplementary file 1 [file ijms-25-00376-s001.zip › ijms-2740297-supplementary.pdf]

Peptidomics Unveils Distinct Acetylation Patterns of Histone and Annexin A1 in Differentiated Thyroid Cancer

Supplementary material

Tables

Table S1. Discrimination of the thyroid nodules of the entire studied population. Diagnosis by fine-needle aspiration biopsy (FNAB) cytology [The 2023 Bethesda System for Reporting Thyroid Cytopathology (Ali, Baloch et al. 2023)] and histology diagnosis [The 2022 WHO Classification of Thyroid Neoplasms (Lloyd, Osamura et al. 2022)] of all samples, including subtypes in parenthesis. Plus, discrimination of the thyroid nodules used for HR-MAS ^{1}H NMR metabolomics. Abbreviations: AUS – atypia of undetermined significance; BT – benign tumours; FTC – follicular thyroid carcinoma; PTC – papillary thyroid carcinoma.

Individual	Sample	Cytology diagnosis	Histology diagnosis	HR-MAS ^{1}H NMR
TR008	A	AUS	BT (Folicular adenoma)	
TR012	A	Benign	BT (Follicular nodular disease)	✓
	B	Benign	BT (Follicular nodular disease)	
TR013	A	Follicular tumour	PTC (Follicular)	✓
TR014	A	Benign	BT (Follicular nodular disease)	
	B		BT (Follicular nodular disease)	
	C		BT (Follicular nodular disease)	
TR015	A		BT (Follicular nodular disease)	
	B		BT (Follicular nodular disease)	
TR018	A	Benign	BT (Follicular nodular disease)	✓
TR019	A	Benign	BT (Follicular nodular disease)	
	B		BT (Follicular nodular disease)	
TR020	A	Follicular tumour (oncocytic)	FTC (Oncocytic)	✓
TR021	A	Benign	BT (Follicular adenoma)	✓
	B	AUS	BT (Follicular nodular disease)	
TR023	A	Benign	BT (Follicular nodular disease)	
TR025	A		BT (Follicular nodular disease)	
	B		BT (Follicular nodular disease)	
	C	Benign	BT (Follicular nodular disease)	
TR026	A	Follicular tumour	BT (Follicular nodular disease)	✓
	B	Benign	BT (Follicular nodular disease)	
TR028	A	Follicular tumour	PTC (Follicular and solid)	✓
TR029	A	Benign	BT (Follicular nodular disease)	
TR030	A		BT (Follicular nodular disease)	
TR031	A	Benign	BT (Follicular nodular disease)	✓
	B	Follicular tumour	BT (Follicular nodular disease)	
TR032	A	Follicular tumour	PTC (Follicular)	✓
TR034	A	Papillary carcinoma	PTC (Classical and follicular)	✓
	B		PTC (Classical and follicular)	
TR035	A	Benign	BT (Follicular nodular disease)	
TR037	A		BT (Follicular nodular disease)	✓
	B		BT (Follicular nodular disease)	
	C		BT (Follicular nodular disease)	
	D		BT (Follicular nodular disease)	
TR038	A	Non-diagnostic	BT (Follicular adenoma)	✓
	B		BT (Follicular nodular disease)	
TR039	A	Benign	BT (Follicular nodular disease)	✓
TR040	A	Benign	BT (Follicular nodular disease)	✓
	B		BT (Follicular nodular disease)	
TR041	A	Benign	BT (Follicular nodular disease)	
TR043	A	Benign	BT (Follicular nodular disease)	
	B		BT (Follicular nodular disease)	
	C	Benign	BT (Follicular nodular disease)	
	D		BT (Follicular nodular disease)	
TR045	A	Non-diagnostic	BT (Follicular nodular disease)	
	B		BT (Follicular nodular disease)	
TR046	A	Benign	BT (Follicular nodular disease)	
	B	Benign	BT (Follicular nodular disease)	
TR047	A		BT (Follicular nodular disease)	✓
	B	Benign	BT (Follicular nodular disease)	
TR048	A	Benign	BT (Follicular nodular disease)	✓
TR049	A	Benign	BT (Follicular nodular disease)	

TR050	A	Papillary carcinoma	PTC (Classical and follicular)	✓
TR051	A	Papillary carcinoma	PTC (Classical)	✓
TR052	A	Benign	BT (Follicular nodular disease)	
	B	Benign	BT (Follicular nodular disease)	
TR053	A	Papillary carcinoma	PTC (Classical)	✓
TR054	A	Papillary carcinoma	PTC (Diffuse sclerosing)	✓
TR055	A	Papillary carcinoma	PTC (Classical)	✓
TR056	A	Benign	BT (Follicular nodular disease)	✓
	B	Benign	BT (Follicular nodular disease)	
TR057	A	Non-diagnostic	BT (Follicular nodular disease)	✓
TR058	A	Non-diagnostic	BT (Follicular nodular disease)	
	B		BT (Follicular nodular disease)	
TR059	A	Benign	BT (Follicular nodular disease)	
TR060	A	Benign	BT (Follicular nodular disease)	
	B	Benign	BT (Follicular nodular disease)	
	C	Benign	BT (Follicular nodular disease)	
TR061	A	Benign	BT (Follicular nodular disease)	
	B	Benign	BT (Follicular nodular disease)	
TR063	A	Oncocytic adenoma	BT (Oncocytic adenoma)	
Individuals 43		Non-diagnostic 4<br>AUS 2	Inadequate or different from cytology	8 (15.7%)

In the sample column different letters represent different nodules studied for the same individual.

Table S2. Top 20 statistically different proteins and peptides with fold-change (FC), p-value from the Mann-Whitney test and adjusted p-value based on FDR (Adj p-value).

Protein	FC	p-value	Adj p-value
P04083|ANXA1	2.44	2.74E-07	1.48E-04
Q9UKK3|PARP4	2.25	5.80E-07	1.48E-04
P08727|K1C19	4.24	5.80E-07	1.48E-04
P50995|ANX11	1.58	5.80E-07	1.48E-04
Q9UPN3|MACF1	1.28	7.41E-07	1.48E-04
Q9NZM1|MYOF	1.44	7.42E-07	1.48E-04
P06753|TPM3	1.7	8.05E-07	1.48E-04
Q15149|PLEC	1.7	8.05E-07	1.48E-04
Q99536|VAT1	1.09	8.05E-07	1.48E-04
P02763|A1AG1	-3.02	8.73E-07	1.48E-04
O60831|PRAF2	1.41	9.08E-07	1.48E-04
Q6UX53|MET7B	4.21	9.46E-07	1.48E-04
Q9BUF5|TBB6	1.35	9.47E-07	1.48E-04
Q14195|DPYL3	2.17	1.11E-06	1.48E-04
Q9BTV4|TMM43	1.95	1.21E-06	1.48E-04
Q9BSJ8|ESYT1	1.36	1.21E-06	1.48E-04
P46821|MAP1B	2.09	1.53E-06	1.48E-04
Q9P2R3|ANFY1	0.92	1.66E-06	1.48E-04
Q13509|TBB3	2.98	1.66E-06	1.48E-04
P02545|LMNA	1.28	1.66E-06	1.48E-04

Peptides	FC	p-value	Adj p-value
P16402|H13|[1Ac]-SETAPLAPTIPAPAEKTPVKK	4.88	1.31E-06	1.16E-04
P10412|H14|SETAPAAPAAPAPAEKTPVK	3.22	2.27E-06	1.16E-04
P08670|VIME|FGGPGTASRPSS	2.59	3.10E-06	1.16E-04
P10412|H14|[1Ac]-SETAPAAPAAPAPAEKTPVKK	3.72	3.10E-06	1.16E-04
P16403|H12|[1Ac]-SETAPAAPAAAPPAEKAPVKK	3.16	6.63E-06	1.98E-04
Q9Y3U8|RL36|ALRYPMAV	2.70	8.29E-06	2.05E-04
O00479|HMGN4|DASTLQSQKAEGTGDAK	2.07	9.62E-06	2.05E-04
Q5VTE0|EF1A3|[1Ac]-GKEKTHINIVVIGHVDSGKST	3.73	1.98E-05	3.69E-04
P16403|H12|[1Ac]-SETAPAAPAAAPPAEK	3.79	2.82E-05	4.32E-04
P10412|H14|[1Ac]-SETAPAAPAAPAPAEKTPVKKK	2.73	3.03E-05	4.32E-04
Q9BX97|PLVAP|GLAMEHGGSYA	-2.47	3.25E-05	4.32E-04
P04083|ANXA1|[1Ac]-AMVSEFLKQ	3.90	3.48E-05	4.32E-04
P16402|H13|[1Ac]-SETAPLAPTIPAPAEKTPVKKK	3.76	3.99E-05	4.55E-04
P10809|CH60|AKDVKFGADARALMLQGVDLLADAVAV	2.61	4.28E-05	4.55E-04
Q5VTE0|EF1A3|IGGIGTVPVGRVETGVLKPG	3.19	5.25E-05	5.21E-04
P02671|FIBA|SQLQKVPPEWKALTDMPQM	-2.60	6.87E-05	5.76E-04
P60709|ACTB|FAGDDAPRAVFPSIVGRPR	4.91	7.35E-05	5.76E-04
P47914|RL29|AAAPASVPAQAPKR	3.15	7.35E-05	5.76E-04
P16402|H13|[1Ac]-SETAPLAPTIPAPAEK	2.71	7.35E-05	5.76E-04
P08729|K2C7|[1Ac]-SIHFSSPVFTSRSA	-2.59	0.000116	8.67E-04

Figures

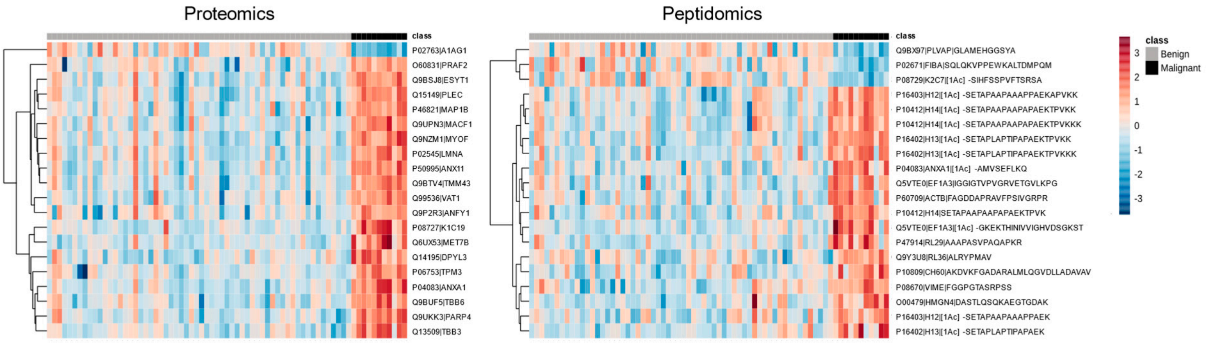

Figure S1. Heatmap with hierarchical clustering of top 20 statistically different proteins (left panel) and peptides (right panel). Euclidean distance measure and ward clustering method. Proteins are represented by their Uniprot accession number and entry name. Peptides are identified by their protein the same way, plus the one code amino acid sequence.

References

Ali, S. Z., Z. W. Baloch, B. Cochand-Priollet, F. C. Schmitt, P. Vielh and P. A. VanderLaan (**2023**). "*The 2023 Bethesda System for Reporting Thyroid Cytopathology.*" Thyroid.

Lloyd, R., R. Osamura and J. Rosai (**2022**). *WHO Classification of Tumours Editorial Board. Endocrine and Neuroendocrine tumours*, Lyon: IARC.
